# The value of deep inspirations

**DOI:** 10.1152/ajplung.00249.2025

**Published:** 2025-08-01

**Authors:** Gary C. Sieck

**Affiliations:** Department of Physiology and Biomedical Engineering, Mayo Clinic, Rochester, Minnesota, United States

Since the initial observations of the Greek physician Erasistratus in the 3rd century BCE, it is recognized that breathing is a motor function that depends on the pumping action of diaphragm muscle contraction (1). However, recognition that lung airflow is regulated by muscle contraction was not considered until the early 19th century. Airway smooth muscle (ASM) was only first described by the German anatomist Franz Reisseisen in the early 1800s (1). The involvement of ASM contraction in asthma was first proposed by Henry Salter (2) in 1868. It is now widely recognized that ASM contraction is a major contributor to the pathophysiology of asthma. However, the exact role ASM contraction in the normal physiological function of breathing remains unclear. It is known that contraction of ASM cells constrict the airway lumen thereby controlling the caliber of airways and the resistance to air flow. Typically, ASM displays a state of spontaneous contraction or “tone” that establishes the basal caliber of the airways (3). Many investigators consider ASM to be vestigial with a physiological function that was lost in evolution. However, the regulation of ASM tone and airway caliber may be important in controlling the distribution of airflow and ventilation in the lungs.

## COUPLING ASM TONE TO THE RESPIRATORY CYCLE

In early physiological studies, it was observed that the bronchial airways dilate (relax) during inspiration and constrict during expiration (4). Following up on this apparent coupling of bronchial airway mechanics and breathing patterns, Melville and Caplan (5) reported that in dogs maximal lung inflation mitigated, at least temporarily, experimentally induced bronchoconstriction. Subsequently, Nadel and Tierney (6) demonstrated a similar effect of deep inspiration in reducing airway resistance in humans. These initial observations led to numerous other studies exploring the effect of deep inspirations on lung and airway mechanics, particularly focusing on the ability (or not) of deep inspirations to dilate already constricted airways or to protect against bronchoconstriction.

The beneficial effect of taking a deep inspiration has an ancient origin in spiritual traditions, especially those of India, Tibet, and China. In these practices, breath control including deep breathing is a core component for improving both physical and spiritual well-being. It is safe to say, that breath control, especially deep inspiration has been recognized for thousands of years for its profound effects on relaxation. As mentioned, in respiratory physiology, the beneficial effect of deep inspiration in relaxing the airway has been recognized for many years. Research over the past 70–80 years has focused on the interplay between deep inspirations, airway and lung mechanics, and airway hyperresponsiveness, especially in the context of asthma.

## EFFECT OF STRAIN ON DYNAMIC PROPERTIES OF CROSS-BRIDGE CYCLING

The mechanisms underlying the coupling of ASM tone to the respiratory cycle and the profound relaxation of ASM tone induced by deep inspirations are not fully understood. Early studies attributed ASM hyperreactivity and bronchoconstriction during asthma to abnormal control by the autonomic nervous system that innervates ASM (7). However, in in vitro preparations such as the ex vivo sheep lung preparation used in this journal by Yasuda et al. (8), any neural mechanism underlying deep inflation-mediated relaxation is excluded. It is likely that the effect of deep inspiration is due to the mechanical strain imposed on the contractile machinery within ASM and the disruption of actin-myosin cross bridges (7, 9). In porcine ASM strips, rapid stretch was shown to break cross-bridge attachments and thereby relax force generation. As cross bridges reform over time, actin filaments must tether to the cortical cytoskeleton of ASM cells to translate force. The tethering of actin filaments and the resulting force generation increases with time, whereas the cross-bridge cycling and ATP consumption rate are affected inversely by the changing internal load on cross bridges. Thus, cross-bridge cycling and ATP consumption rate are initially higher whereas external force generation and internal load imposed by the tethering on cross bridges are lower (9, 10). Thus, mechanical strain on ASM has a marked effect on fundamental dynamic properties of cross-bridge cycling kinetics, force generation, relaxation, and energetics (9, 10).

## DYNAMIC EQUILIBRIUM: HOMEOSTASIS OF BRONCHOMOTOR TONE

Walter Cannon introduced the concept of homeostasis not as a constant physiological state but as a dynamic equilibrium in which the body adjusts to internal or external perturbations to keep physiological parameters in a normal healthy range. Although ASM and bronchomotor tone may be vestigial, it is subject to homeostasis and dynamic adjustments. Although not explicitly stated, the recent paper by Yasuda et al. (8), used an ex vivo sheep lung model to explore homeostasis of bronchomotor tone during low tidal volume (lower strain on ASM) ventilation and increased lung inflation (mimicking deep inspiration and higher strain). In their study, they observed a spontaneous increase in bronchomotor tone and airway resistance that occurs with low tidal volume ventilation. This spontaneous increase in bronchomotor tone during low tidal volume (low strain) ventilation results in the closing (due to constriction) of airways and air trapping—common features of asthma. Thus, without higher strain perturbations, ASM force generation increases progressively due to increasing cross-bridge formation. They hypothesized that deep inspirations perturbs the spontaneous increase in ASM force generation (bronchomotor tone) during low tidal volume ventilation. Thus, the spontaneous increase in ASM contraction continued when deep inspirations were prevented, which allowed “basal airway smooth muscle (ASM) tone to narrow and close airways over time, resulting in elevation of airway and lung resistance, as well as air trapping.” The bronchodilatory effects of deep inspirations that they observed were compared with those induced by salbutamol-induced relation of ASM force generation. With both deep inspirations and pharmacological inhibition of ASM contraction, the spontaneous increase in airway and lung resistance and air trapping with low tidal volume ventilation were reduced. The results of this study clearly demonstrated the benefit and requirement of deep inspirations in reducing spontaneous bronchoconstriction. Intermittent deep inspirations (sighs) are necessary to prevent the progressive increase in bronchomotor tone and the consequent air trapping. Thus, deep inspirations are necessary to maintain normal lung mechanics and function. In addition to providing insight into the dynamic equilibrium of bronchomotor tone, the ventilated ex vivo sheep lung preparation provides an important model to explore both the normal physiology of the lung and the pathophysiology of asthma.

## References

[1] Otis AB. A perspective of respiratory mechanics. J Appl Physiol Respir Environ Exerc Physiol 54: 1183–1187, 1983. doi:10.1152/jappl.1983.54.5.1183. 6345491

[2] Salter HH. On Asthma: Its Pathology and Treatment. John Churchill & Sons, 1868, p. 24–60.

[3] Wylam ME, Xue A, Sieck GC. Mechanisms of intrinsic force in small human airways. Respir Physiol Neurobiol 181: 99–108, 2012. doi:10.1016/j.resp.2012.01.011. 22322114 PMC4066449

[4] Ellis MP. The mechanism of the rhythmic changes in the calibre of the bronchi during respiration. J Physiol 87: 298–309, 1936. doi:10.1113/jphysiol.1936.sp003408. 16994796 PMC1395246

[5] Melville KI, Caplan H. The influence of lung distension upon the response of the bronchioles to epinephrine and histamine. J Pharmacol Exp Ther 94: 182–191, 1948. 18889412

[6] Nadel JA, Tierney DF. Effect of a previous deep inspiration on airway resistance in man. J Appl Physiol 16: 717–719, 1961. doi:10.1152/jappl.1961.16.4.717. 13727342

[7] Seow CY, Fredberg JJ. Historical perspective on airway smooth muscle: the saga of a frustrated cell. J Appl Physiol (1985) 91: 938–952, 2001. doi:10.1152/jappl.2001.91.2.938. 11457813

[8] Yasuda Y, Maksym GN, Wang L, Chitano P, Seow CY. Low tidal volume ventilation facilitates spontaneous increase in bronchoconstriction and air trapping that can be resolved by deep inspiration and bronchodilator. Am J Physiol Lung Cell Mol Physiol 329: L225–L233, 2025. doi:10.1152/ajplung.00085.2025. 40590786

[9] Fredberg JJ, Jones KA, Nathan M, Raboudi S, Prakash YS, Shore SA, Butler JP, Sieck GC. Friction in airway smooth muscle: mechanism, latch, and implications in asthma. J Appl Physiol (1985) 81: 2703–2712, 1996. doi:10.1152/jappl.1996.81.6.2703. 9018525

[10] Delmotte P, Han YS, Sieck GC. Cytoskeletal remodeling slows cross-bridge cycling and ATP hydrolysis rates in airway smooth muscle. Physiol Rep 8: e14561, 2020. doi:10.14814/phy2.14561. 32812390 PMC7435030

